# The schizophrenia risk gene *Map2k7* regulates responding in a novel contingency-shifting rodent touchscreen gambling task

**DOI:** 10.1242/dmm.049310

**Published:** 2022-03-11

**Authors:** Rebecca L. Openshaw, Judith A. Pratt, Brian J. Morris

**Affiliations:** 1 Institute of Neuroscience and Psychology, College of Medical, Veterinary and Life Sciences, University of Glasgow, Glasgow G12 8QQ, UK; 2 Strathclyde Institute of Pharmacy and Biomedical Sciences, University of Strathclyde, Glasgow G4 0RE, UK

**Keywords:** Schizophrenia, Genetic risk, Anxiety, Cognition, Decision making

## Abstract

In schizophrenia, subjects show reduced ability to evaluate and update risk/reward contingencies, showing correspondingly suboptimal performance in the Iowa gambling task. JNK signalling gene variants are associated with schizophrenia risk, and JNK modulates aspects of cognition. We therefore studied the performance of mice hemizygous for genetic deletion of the JNK activator MKK7 (*Map2k7*^+/−^ mice) in a touchscreen version of the Iowa gambling task, additionally incorporating a novel contingency-switching stage. *Map2k7*^+/−^ mice performed slightly better than wild-type (WT) littermates in acquisition and performance of the task. Although *Map2k7*^+/−^ mice adapted well to subtle changes in risk/reward contingencies, they were profoundly impaired when the positions of ‘best’ and ‘worst’ choice selections were switched, and still avoided the previous ‘worst’ choice location weeks after the switch. This demonstrates a precise role for MKK7–JNK signalling in flexibility of risk/reward assessment and suggests that genetic variants affecting this molecular pathway may underlie impairment in this cognitive domain in schizophrenia. Importantly, this new contingency shift adaptation of the rodent touchscreen gambling task has translational utility for characterising these cognitive subprocesses in models of neuropsychiatric disorders.

## Abstract (precis)

**Summary:** This work identifies in mice a single, specific gene variation linked to schizophrenia risk (hemizygosity for genetic deletion of MKK7) that produces a specific cognitive impairment exhibited by patients with schizophrenia.

## INTRODUCTION

The ability to make sound decisions, incorporating aspects of emotion, memory of past events and projections to future events, is a crucial element of normal cognitive function. Subjects with schizophrenia have altered decision-making abilities (Fond et al., 2013), demonstrated using the Iowa gambling task (IGT) (Bechara et al., 1994). The IGT involves probabilistic learning via monetary reward and punishment, with the subject choosing cards from four decks. Some decks are advantageous in the short term (large wins) but disadvantageous in the long run (large, frequent losses); other choices are less attractive in the short term (small wins), but advantageous over time (small, less frequent losses). Advantageous task performance requires subjects to sacrifice potential large, immediate rewards for smaller rewards, to avoid larger overall losses.

Subjects with schizophrenia make more disadvantageous decisions than healthy controls in the IGT (Shurman et al., 2005; Kester et al., 2006; Turnbull et al., 2006; Sevy et al., 2007; Kim et al., 2009; 2016; Cella et al., 2012; Brambilla et al., 2013; Fond et al., 2013; Pedersen et al., 2017; Betz et al., 2019; Woodrow et al., 2019), although occasional studies have failed to detect deficits (Evans et al., 2005; Rodríguez-Sánchez et al., 2005), possibly reflecting the heterogeneity of schizophrenia (Beninger et al., 2003; Bark et al., 2005). Turnbull et al. (2006), created an IGT version in which reward/punishment contingencies associated with each deck were changed during the session, thus probing adaptive decision making. Patients with schizophrenia who had high negative symptom scores, despite performing as well as controls in learning the basic IGT, showed difficulty in shifting responses when the reward/punishment contingencies of the cards were reversed. This may be perseverative behaviour, with patients either over-relying on previously favourable decks, or else persistently avoiding decks previously learned to be unfavourable (Turnbull et al., 2006).

Rodent cognitive tasks based on touchscreen hardware have been developed to maximise translational validity with corresponding human tasks (Bartko et al., 2011; Bussey et al., 2012; Nilsson et al., 2016a; 2016b; 2018; Wulaer et al., 2018; Zeleznikow-Johnston et al., 2018; Liao et al., 2022). A rodent version of the IGT (rGT; reviewed in van den Bos et al., 2014) has been validated for cross-species investigation of cognition within the Research Domain Criteria (RDoC)/Cognitive Neuroscience Treatment Research to Improve Cognition in Schizophrenia (CNTRICS) cognitive constructs (Cope et al., 2016). In a similar way to the IGT, the rGT presents rodents with four options on a touchscreen that differ in frequency and magnitude of reward/punishment possibilities. Rats (Zeeb et al., 2009) and mice (van Enkhuizen et al., 2013) consistently favour the advantageous choices versus the disadvantageous choices in the corresponding five-hole operant box version. Neural circuitry in both rodent and human versions of the gambling task are similar, requiring prefrontal cortex, orbitofrontal cortex, striatum and amygdala (de Visser et al., 2011).

There is a major genetic component to schizophrenia risk, reflecting the action of hundreds of common variants, each individually of small effect, or small numbers of rare variants with much greater individual effect (Owen et al., 2010; Mulle, 2012). The polygenic nature of risk suggests that clusters of functionally related genes will act synergistically to perturb central nervous system function, and, indeed, multiple risk loci are predicted to affect glutamatergic synapses (Fromer et al., 2014; Purcell et al., 2014; Marshall et al., 2017; Rees et al., 2019) and JNK signalling (Winchester et al., 2012; Kirov et al., 2014; Morris and Pratt, 2014; Rees et al., 2014; Ripke et al., 2020). Abundant evidence links glutamatergic synapse function to cognitive processes, and accumulating evidence additionally implicates JNK signalling in cognition, particularly in salience-dependent aspects of associative learning (Kenney et al., 2010; Sherrin et al., 2010; Leach et al., 2015; Morel et al., 2018).

Mitogen-activated protein kinase kinase 7 (MKK7) is one of two upstream kinases involved in JNK activation. We previously reported that mice hemizygous for deletion of the gene encoding MKK7, *Map2k7* (*Map2k7^+/−^* mice), show impaired levels of attention in a reward-based five-choice task (Openshaw et al., 2016). However, it is not known whether JNK activation is important in reward-based or adaptive decision making. In this study, we characterise the performance of *Map2k7^+/−^* mice and their wild-type (WT) littermates in the rGT. Importantly, we have adapted the task to investigate performance when reward/punishment contingencies are reversed, thereby interrogating flexibility of adaptive decision making.

## RESULTS

### *Map2k7*^+/−^ mice show slightly less-risky choice behaviour in the rGT

#### Choice performance

**Fig. 1.**
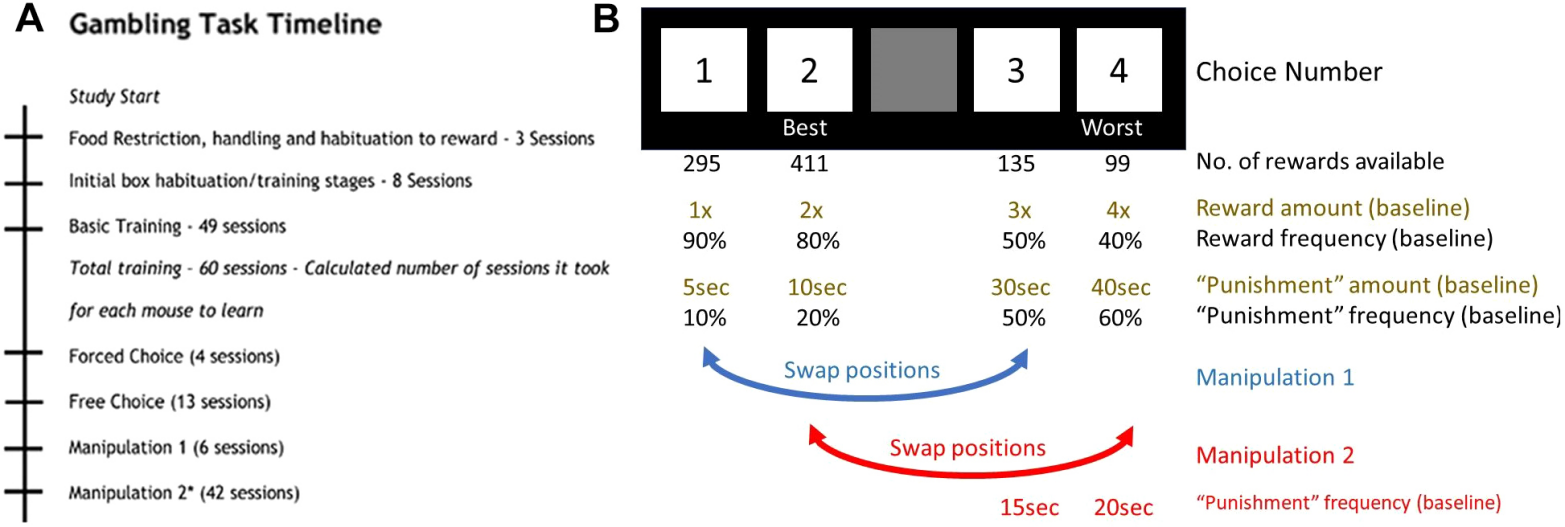
**Experimental timeline and illustration of risk/reward contingencies used.** (A) Timeline showing the experimental procedure. (B) Schematic depicting the reward/punishment contingencies associated with each choice on the touchscreen. Choice 2 is the most advantageous option and Choice 4 is the least advantageous, according to the number of reward deliveries available per session. Taking into account the probabilities and extent of the ‘punishment’ (timeout with light on) period, if chosen exclusively within the 30 min period, it is possible for mice to obtain 295 reward deliveries with Choice 1, 411 with Choice 2, 135 with Choice 3 and 99 with Choice 4. In Manipulation 1, the locations of Choices 1 and 3 were switched. In Manipulation 2, the locations of Choices 2 and 4 were switched, and the punishment periods for Choices 3 and 4 were decreased.

**Fig. 2.**
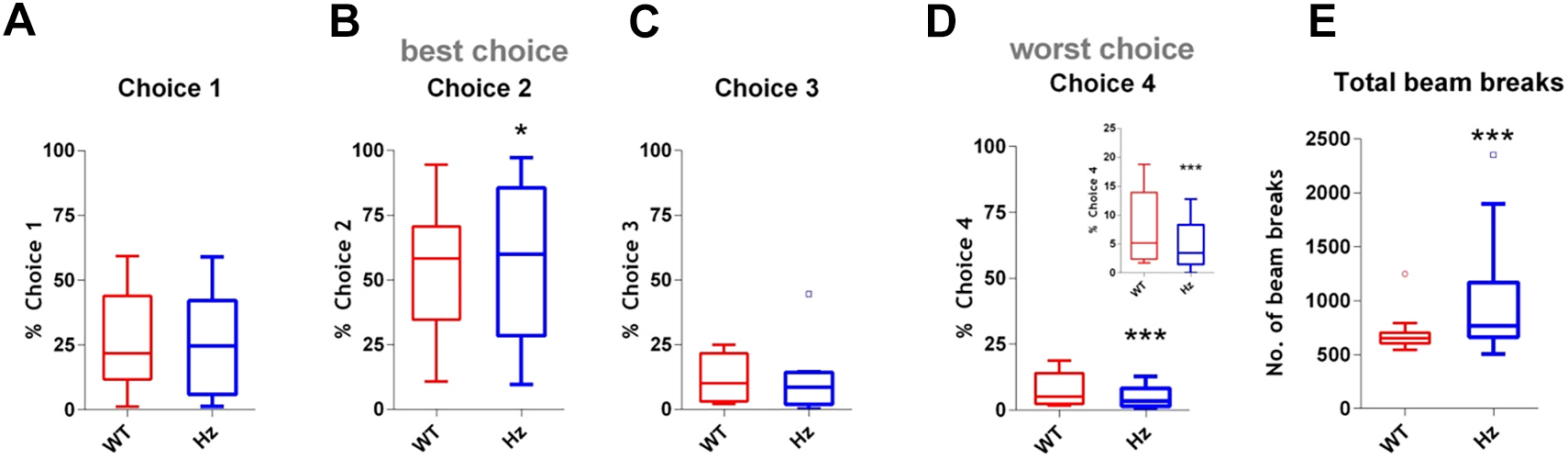
***Map2k7^+/−^* mice show less-risky choice selection, and hyperactivity, in the rGT.** *Map2k7^+/−^* (Hz) mice show enhanced performance compared to wild-type (WT) mice. (A-D) *Map2k7^+/−^* mice selected similar numbers of Choices 1 (A; genotype effect *P*=0.795) and 3 (C; genotype effect *P*=0.237) as WT mice, but made significantly more selections of Choice 2 [B; the ‘most optimal’ choice, *P=0.011, *F*_(1,80)_=6.73] and significantly fewer selections of Choice 4 [D; the ‘least optimal’ choice, ***P<0.0001, *F*_(1,80)_=15.85]. (E) *Map2k7^+/−^* mice were more active than WT mice, making more beam breaks [***P<0.0001, *F*_(1,80)_=461.80]. Box plots show median and interquartile range, with Tukey whiskers; outliers are represented by dots. Data analysed by three-way repeated measures ANOVA with the last 5 days’ session at baseline as a within-subjects factor, and genotype and sex as between-subjects factors. Inset in D shows the same data, but with an expanded ordinate axis for clarity.

**Fig. 3.**
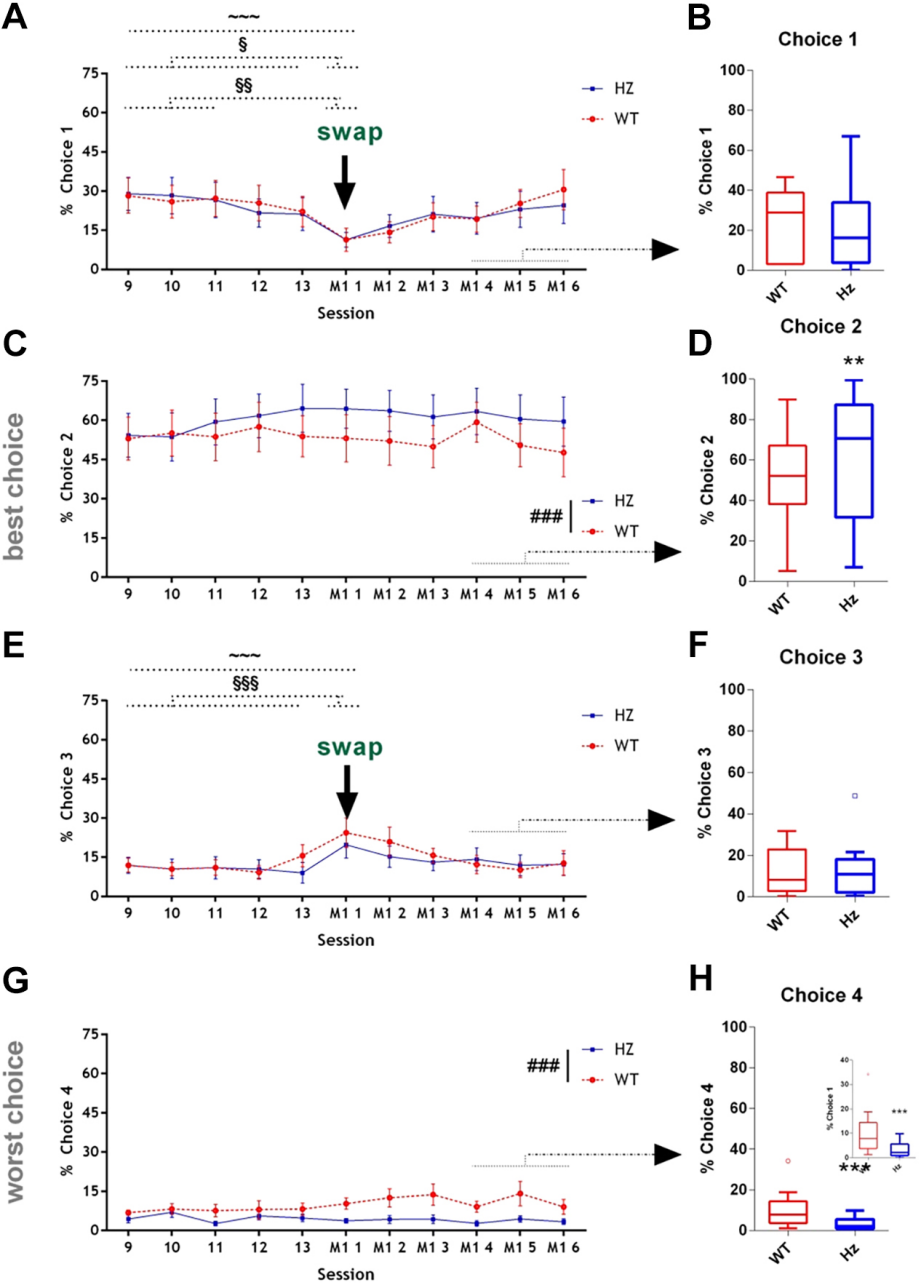
**rGT performance was affected by Manipulation 1 to the same extent in WT and *Map2k7^+/−^* mice.** The locations of intermediate Choices 1 and 3 were swapped when indicated by the arrow. (A,C,E,G) Line graphs show % selection of each choice for the last 5 days of baseline testing (days 9-13, equivalent to data shown in Fig. 2), and the first 6 days following Manipulation 1. Comparing the first day of Manipulation 1 to the last 5 days’ stable responding revealed a significant effect of the manipulation on selection of Choices 1 [A; ^∼∼∼^*P*<0.0001, *F*_(5,131)_=6.71] and 3 [E; ^∼∼∼^*P*<0.0001, *F*_(5,131)_=7.42]. There was no significant change for Choices 2 (C; *P*=0.132) and 4 (G; *P*=0.315). Data points represent the mean; error bars represent the s.e.m. ^§^*P*<0.05 versus each of days 9-13; ^§§^*P*<0.001 versus each of days 9-11; ^§§§^*P*<0.001 versus each of days 9-13, Tukey's post hoc test. ^###^*P*<0.0001, significant main effects of genotype [session (last 5 days, plus first day of Manipulation 1)]. Choice 2 [C; *P*<0.0001, *F*_(1,131)_=11.00]; Choice 4 [G; *P*<0.0001, *F*_(1,131)_=26.33]. (B,D,F,H) Analysing the last three sessions of Manipulation 1 (days 4-6) showed that *Map2k7^+/−^* mice were similar to WTs with their responses to Choices 1 (B; *P*=0.325) and 3 (F; *P*=0.478), but *Map2k7^+/−^* mice responded significantly more than WTs to Choice 2 [D; *P*=0.001, *F*_(1,40)_=12.47] and less to Choice 4 [H; *P*<0.001; *F*_(1,40)_=56.28]. Inset in D shows the same data, but with an expanded ordinate axis for clarity. Box plots show median and interquartile range, with Tukey whiskers; outliers are represented by dots. Data analysed by three-way repeated measures ANOVA with session (last 3 days following Manipulation 1) as a within-subjects factor, and genotype and sex as between-subjects factors. **P<0.01, ***P<0.0001.

Mice progressed through initial training and then were trained on the free-choice rGT until they reached stable choice performance, which took 13 sessions in total. The timeline followed, and the risk/reward contingencies associated with the different choice options, are shown in Fig. 1. Data for the performances of both groups during the acquisition stage of the task are shown in Table S1. The last 5 days of stable performance (referred to here as ‘baseline’) were then analysed for differences between genotypes. *Map2k7^+/−^* mice showed enhanced performance on the rGT at baseline compared to WTs, with decreased % Choice 4 (the least optimal option), and a slight increase in responding for the most optimal choice, Choice 2 (Fig. 2B,D). The % selection of the intermediately advantageous Choices 1 (Fig. 2A) and 3 (Fig. 2C) were similar between WT and *Map2k7^+/−^* mice.

The *Map2k7^+/−^* mice were also more active than WT mice, generating more beam breaks (Fig. 2E). They collected the reward just as quickly as WT mice [genotype effect *P*=0.138, three-way repeated-measures ANOVA, non-significant (ns); Table S1]; however, they were quicker to make a choice [*P*<0.0001, *F*_(1,80)_=34.96, three-way repeated-measures ANOVA; Table S1]. WT and *Map2k7^+/−^* mice made similar % omissions (*P*=0.192, three-way repeated-measures ANOVA, ns; Table S1) and % premature responses (see Fig. 5C).

Overall, *Map2k7^+/−^* mice seemed highly motivated to complete the rGT. They were hyperactive and faster to make a choice, and were able to select a slightly higher proportion of the most optimal choice and a slightly lower proportion of the least optimal choice.

### Manipulation 1: switching reward/punishment contingencies of most similar optimal choices affects performance in the rGT to the same extent in WT and *Map2k7*^+/−^ mice

Manipulation 1 involved a switch between options 1 and 3 (see Fig. 1B), which are the two more subtly different options. As expected, overall performance for all mice during the first session following this manipulation dropped, reflected temporarily decreased selection of Choice 1, and increased selection of Choice 3, because they had switched location (Fig. 3A,E). % Choices for 2 and 4, which were not switched, remained unchanged (Fig. 3C,G). The increased selection of Choice 2 and reduced selection of Choice 4 by *Map2k7^+/−^* mice was maintained throughout Manipulation 1 (Fig. 3C,D,G,H).

Remarkably, the mice detected the manipulation and rapidly altered their responding. By the time stable performance had been achieved for three consecutive sessions (which took only six sessions in total), the mice had returned to the same pattern of responding as before. Comparing the last 3 days of Manipulation 1 between genotypes showed that *Map2k7^+/−^* mice still had enhanced performance compared to WTs: slightly increased responding for Choice 2 (Fig. 3D), decreased responding for Choice 4 (Fig. 3H) and similar responding for the switched choices, Choices 1 and 3 (Fig. 3B,F).

Over the last 3 days of testing after Manipulation 1, *Map2k7^+/−^* mice made fewer % omissions than WT mice [*P*=0.001, *F*_(1,40)_=13.92, Table S1], a parameter that had previously been similar between genotypes at baseline. All other performance and motivation parameters remained similar between genotypes to what they had been at baseline. Total beam breaks [*P*<0.0001, *F*_(1,40)_=166.16; Table S1] and number of choices made [*P*<0.0001, *F*_(1,40)_=23.24; Table S1] were increased in the *Map2k7^+/−^* mice compared to WTs; *Map2k7^+/−^* mice were quicker to make a choice [*P*<0.0001, *F*_(1,40)_=24.82; Table S1].

### Performance of *Map2k*7^+/−^ mice is greatly impaired when the most and least optimal choices are reversed in Manipulation 2

Manipulation 2 consisted of switching the most versus least optimal options: Choice 2 with Choice 4. The severity of the punishment period was also decreased for Choice 3 and Choice 4, reducing them from 30 s and 40 s to 15 s and 20 s, respectively, while keeping the punishment likelihood the same for all options. The punishment period length now increased linearly from Choices 1 to 4 (from 5 to 10 to 15 to 20 s, respectively), as opposed to increasing disproportionately. Throughout Manipulation 1, the mice were capable of tracking even subtly different contingencies; switching two more obviously different contingencies would likely have the effect of inducing just as much, or even more, motivation to switch because the consequences are more drastic. However, the decrease in the punishment period for the disadvantageous options at the same time as this switch makes this re-evaluation more complex, and enables examination of the magnitude of effect that lesser punishment has on the mice when attempting to learn a new contingency switch, as opposed to other aspects of the task (reward amount, punishment probability), because these remain the same, just in a different location on the touchscreen.

**Fig. 4.**
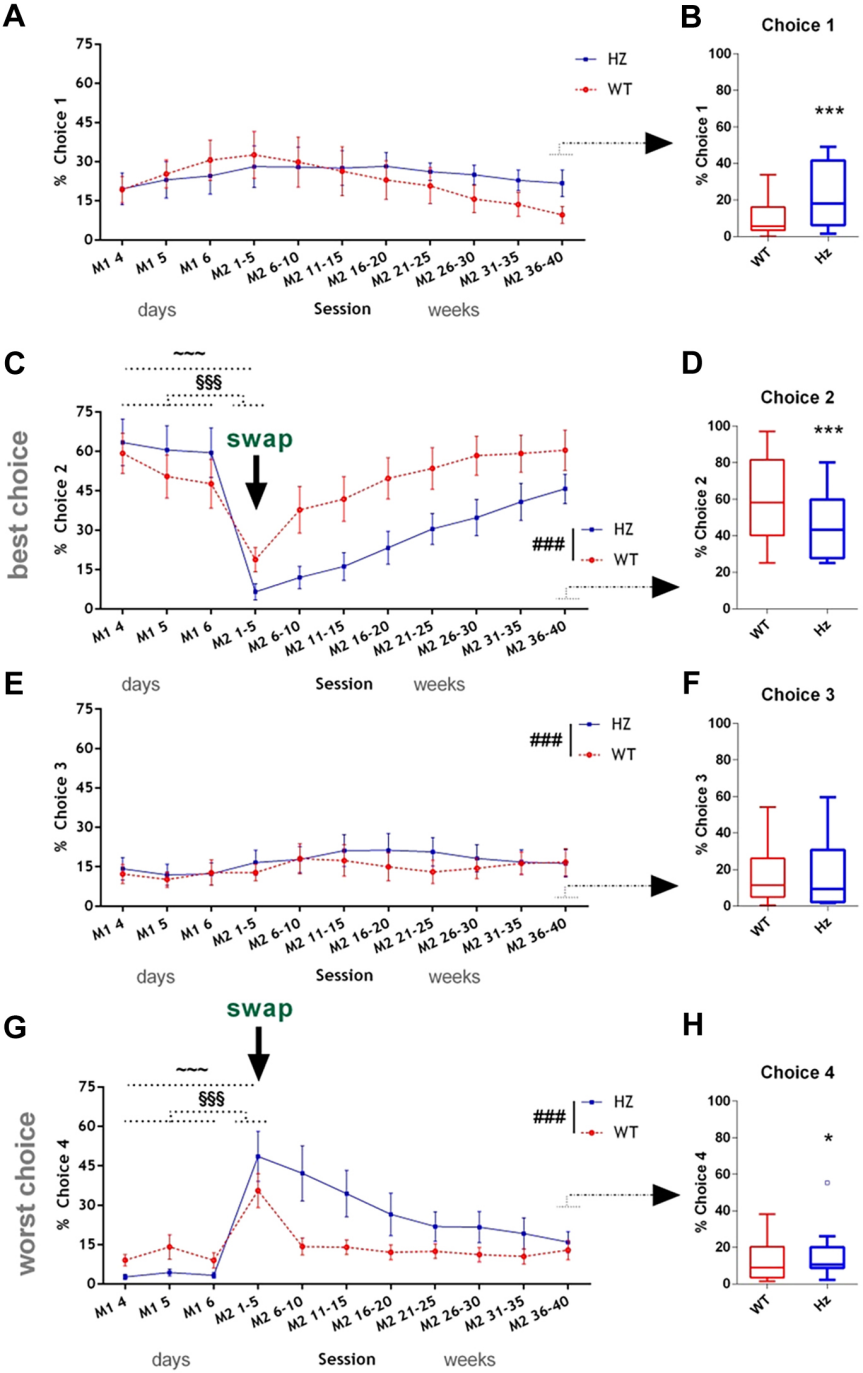
***Map2k7*^+/−^ mice exhibited deficits in adapting to reversing reward–punishment contingencies of the most versus least optimal choices in Manipulation 2.** The locations of best and worst Choices 2 and 4 were swapped when indicated by the arrow, and ‘punishment’ time was also decreased for Choices 3 and 4. (A,C,E,G) Line graphs show % selection of each choice for the last 3 days of testing after Manipulation 1 (M1 4-6, equivalent to data shown in Fig. 3B,D,F,H), and the 40 sessions following Manipulation 2, in blocks of five sessions for clarity. Comparing the first 5 days of Manipulation 2 to the last 3 days’ stable responding after Manipulation 1 revealed a highly significant effect of session, with decreases for Choice 2 [C; ^∼∼∼^*P*<0.0001, *F*_(7,175)_=32.99] and increases for Choice 4 [G; ^∼∼∼^*P*<0.0001, *F*_(7,175)_=23.57]. There were also small, but significant, increases in selection of Choices 1 [A; *P*=0.005, *F*_(7,175)_=3.06] and 3 [E; *P*=0.004, *F*_(7,175)_=3.16]. Data analysed by three-way repeated measures ANOVA with session (last 3 days of Manipulation 1, plus first 5 days of Manipulation 2) as a within-subjects factor, and genotype and sex as between-subjects factors. Genotype × session interactions were also detected for Choice 2 [C; *P*=0.033, *F*_(1,175)_=32.99] and for Choice 4 [G; *P*=0.001, *F*_(7,175)_=11.52]. Data points represent the mean; error bars represent the s.e.m. ^§§§^*P*<0.001 for each of the first 5 days of manipulation 2 (M2 1-5) versus each of last 3 days of Manipulation 1 (M1 4-6), Tukey's post hoc test. Significant main effects of genotype were also detected after Manipulation 2 (first 25 sessions of Manipulation 2, M1-M25, ^###^*P*<0.0001). Choice 2 [C; *P*<0.0001, *F*_(1,175)_=476.46]; Choice 3 [E; *P*<0.0001, *F*_(1,175)_=25.42]; Choice 4 [G; *P*<0.0001, *F*_(1,175)_=253.98]. (B,D,F,H) At stable performance following Manipulation 2 (sessions 36-40), *Map2k7^+/−^* mice made more % Choice 1 (B; *P*<0.0001, *F*_(1,80)_=79.39) (B) and % Choice 4 (H; *P*=0.039, *F*_(1,80)_=4.38), less % Choice 2 (D; *P*<0.0001, *F*_(1,80)_=61.05) and similar % Choice 3 (F; *P*=0.771, non-significant) compared to WTs. Data analysed by three-way repeated measures ANOVA with session as a within-subjects factor, and genotype and sex as between-subjects factors. Significant differences indicated by *P<0.05, ***P<0.0001.

Following switching of reward/punishment contingencies of the most versus least optimal choices (Manipulation 2), a large effect on choice performance was seen in both genotypes, which was exacerbated in *Map2k7^+/−^* mice (Fig. 4). When the last 3 days of Manipulation 1 were compared with the first 5 days of Manipulation 2, for both Choice 2 and 4 there was a highly significant effect of session, as mice adjusted to the new risk/reward balances associated with these 2 locations (Fig. 4C,G). The % response for Choices 1 and 3 also changed (increased) following Manipulation 2, but not to the same extent as with Choices 2 and 4, probably reflecting some reduced capacity for decision making as to which option to select (Fig. 4A,E). The subtly increased selection of Choice 3 may reflect detection of the reduced ‘punishment’ period.

Importantly, genotype influenced adaptation to the altered risk/reward contingencies. *Map2k7^+/−^* mice showed an impaired ability to adapt to the reversal of these contingencies. In comparison to WT mice, they made reduced selections of Choice 2 in its new location (where the ‘worst’ choice had previously been located; Fig. 4C) and correspondingly made more selections of Choice 4 in its new location (Fig. 4G) (where the ‘best’ choice had previously been located).

At stable performance following Manipulation 2 (more than 35 sessions later), *Map2k7^+/−^* mice made more selections of Choice 1 and Choice 4, and fewer selections of Choice 2 (with similar selection of Choice 3) compared to WTs (Fig. 4B,D,F,H). Interestingly, in contrast to what had been observed at baseline, *Map2k7^+/−^* mice were now consistently making more disadvantageous choices than WTs.

**Fig. 5.**
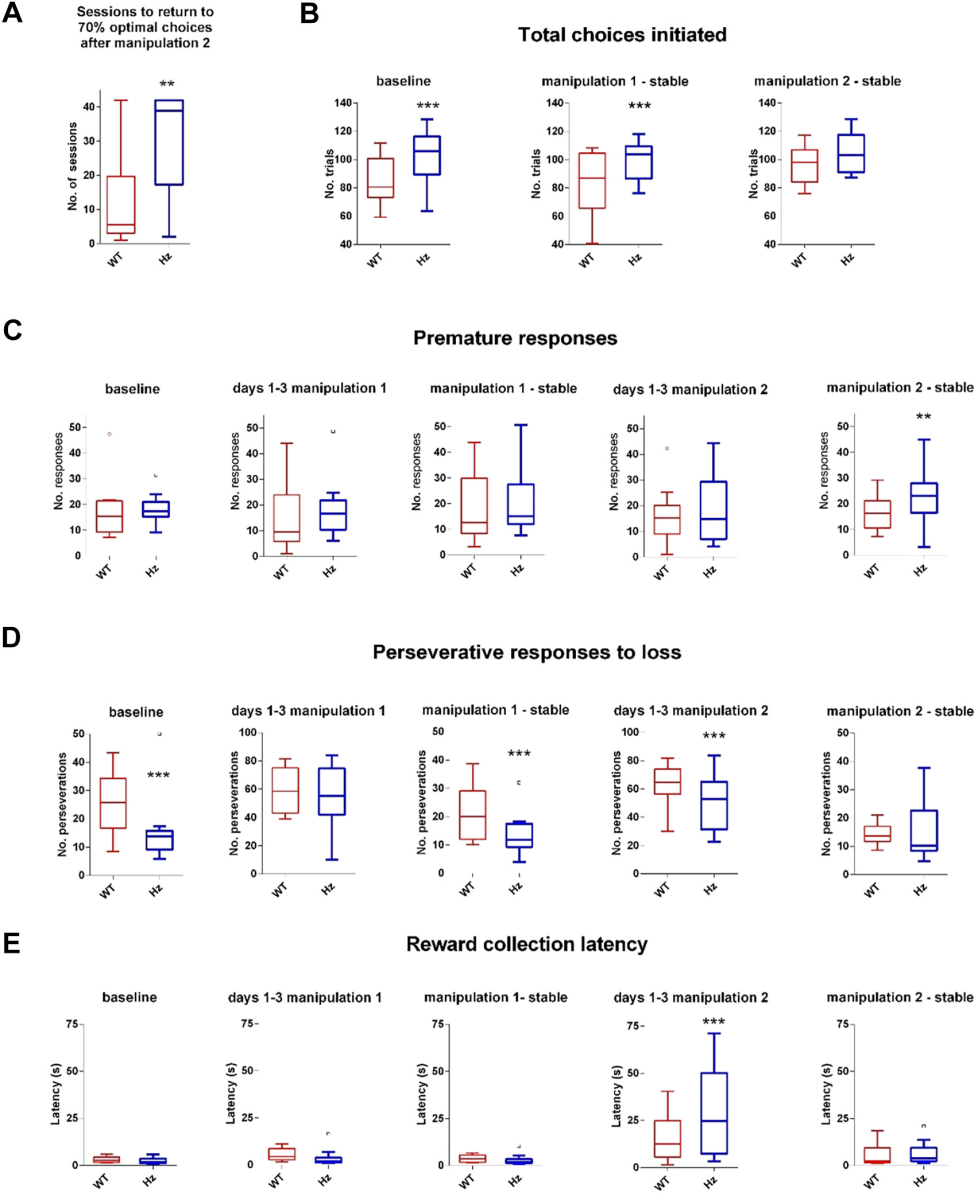
**(A) *Map2k7^+/−^* mice took substantially longer to re-establish stable responding following Manipulation 2.** (A) The number of sessions that it took for each mouse to reach more than 70% of their choices being either Choice 1 or Choice 2 (the two optimal choices) for three consecutive sessions were calculated. Box plots show median and interquartile range, with Tukey whiskers; outliers are represented by dots. Data analysed by one-way repeated measures ANOVA. **P=0.007; *F*_(1,20)_=9.05. (B-E) Total choices initiated (B), premature responses (C), perseverative responses (D) and reward collection latencies (E) are shown at baseline, for the first three sessions after Manipulation 1, once responding is stable after Manipulation 1, over the first three sessions after Manipulation 2 and once responding is stable after Manipulation 2. (B) Choices per session: significant genotype effects were observed at baseline [*P*<0.0001, *F*_(1,80)_=19.16], after Manipulation 1 [*P*<0.0001, *F*_(1,65)_=23.24], but not after Manipulation 2 (*P*=0.928). (C) Premature responses: significant genotype effects were not observed at baseline (*P*=0.651), or after manipulation 1 (*P*=0.108), but were observed once performance had stabilised after Manipulation 2 [*P*=0.001, *F*_(1,80)_=12.98]. (D) Perseverative responses: significant genotype effects were observed at baseline [*P*<0.0001, *F*_(1,80)_=50.53], once performance had stabilised after Manipulation 1 [*P*<0.001, *F*_(1,65)_=27.43] and over the first 3 days of Manipulation 2 [*P*<0.001, *F*_(1,65)_=14.75], but not once performance had stabilised after manipulation 2 (*P*=0.328). (E) Reward collection latency: significant genotype effects were not observed at baseline or once performance had stabilised after the manipulations, but were observed over the first 3 days of Manipulation 1, with *Map2k7^+/−^* mice faster than WTs [*P*=0.007, *F*_(1,65)_=8.11], and over the first 3 days of Manipulation 2, with *Map2k7^+/−^* mice slower than WTs [*P*<0.001, *F*_(1,65)_=28.02]. **P<0.01, ***P<0.001 (genotype main effect, three-way repeated-measures ANOVA).

Other performance parameters were little affected by Manipulation 2. Comparing the first 5 days of Manipulation 2 with the last 3 days of Manipulation 1, % omissions, number of choices made and choice latency were all unaltered (Table S1; *P*=0.573, *P*=0.586, *P*=0.237, respectively). The number of perseverative responses decreased slightly, and % premature responses increased slightly (Fig. 5C,D; *P*=0.007, *P*=0.020, respectively). However, Manipulation 2 had a particularly large effect to increase reward collection latency, for mice of both genotypes, but with a greater effect on *Map2k7^+/−^* mice, which were substantially slower than WT mice (Fig. 5E). This is interesting as, immediately after Manipulation 1, *Map2k7^+/−^* mice were faster than WTs on reward collection latency (Fig. 5E).

All mice appeared to show high motivation throughout Manipulation 2. Aside from the increase in reward collection latency (Fig. 5E), all motivation and other performance parameters changed or remained the same in a way that would, if anything, indicate increased drive to perform [the number of choices made (Fig. 5B), magazine entries and beam breaks increased (Table S1), the % premature responses remained similar (Fig. 5C), and % omissions (Table S1), number of perseverative responses (Fig. 5D) and latency to make a choice (Table S1) were mostly decreased].

Overall, it took mice considerable time to adjust to this dramatic shift in contingencies, and *Map2k7^+/−^* mice, in particular, were never really able to return to their previous levels of performance. *Map2k7^+/−^* mice took substantially longer to re-establish stable responding following this manipulation: 30.25±4.17 sessions to stable performance compared with 12.1±4.32 sessions for WTs (Fig. 4, Fig. 5A).

Evidence of hyperactivity, in terms of a higher number of total beam breaks, was evident in *Map2k7*^+/−^ mice at all stages of the task. We did observe some significant effects of sex, in various parameters of performance (Table S2). No consistent patterns in the sex differences were observed, although female mice tended to be slightly better than males at adjusting back to Choice 2 after Manipulation 2, and also tended to show evidence of being slightly more impulsive and hyperactive than males. However, it should be noted that the study was underpowered to detect genotype×sex interactions.

The results from a number of other behavioural parameters at each stage of the task are shown in Table S1.

In addition, we tested both genotype groups for response to a low dose (1.5 mg/kg) of amphetamine. Overall, amphetamine increased preference for Choice 1 (Fig. S1). The only evidence for differential responses between the genotypes was where WT mice appeared to have greater reactions to amphetamine than *Map2k7*^+/−^ mice in magazine entries during the intertrial interval and choice response latency (Fig. S2).

## DISCUSSION

In this study, we aimed to assess the contribution of MKK7 to reward-related decision making in a rodent touchscreen version of the human IGT, additionally incorporating a novel contingency-switching stage to assess flexibility in risk/reward assessment. We demonstrate that mice show a consistent pattern of optimal responding: avoiding high-risk, high-reward options more than the advantageous options of frequent, small rewards. This is one of the first demonstrations that the four-choice rGT can provide a pattern of responding paralleling that in the human IGT task (Elsilä et al., 2020; Thomson et al., 2021). Here, we show that *Map2k7*^+/−^ mice performed at slightly better levels than WT littermates in acquisition and performance of the task, suggesting that they were able to learn associations of different reward contingencies. We also observe subtle effects of amphetamine administration, consistent with previous reports in equivalent tests in mice and rats (Zeeb et al., 2009; van Enkhuizen et al., 2013; Silveira et al., 2015). When we probed the ability of mice to switch choice selections in a new contingency shift adaption of the task, we showed that *Map2k7*^+/−^ mice adapted to subtle changes in risk/reward contingencies as effectively as control mice, but that they were profoundly impaired when the positions of ‘best’ and ‘worst’ choice selections were switched, and that they perseverated on this behaviour for several weeks. These data support a role for MKK7 in flexible risk/reward assessment akin to findings in schizophrenia, in which in shift phases of the IGT, subjects retain a preference for choices that had previously been ‘good’ even when they experience large losses (Turnbull et al., 2006).

### Choice preference

Overall, all mice showed a choice preference in this touchscreen task that remarkably corresponds with the total possible amounts of reward available, consistent with mouse performance in the nine-hole operant box version of the rGT, using the same reward/punishment contingencies (Zeeb and Winstanley, 2013; Zeeb et al., 2013). Once stable responding was established, *Map2k7^+/−^* mice showed slightly enhanced ability to perform the task: they had a slight preference for Choice 2 and a decreased preference for Choice 4 compared to WT mice. *Map2k7^+/−^* mice may be particularly sensitive to the long punishment period associated with Choice 4, and hence select this option less.

### Motivation performance

Overall, *Map2k7^+/−^* mice were highly motivated to perform: they collected the reward just as quickly as WT mice, were quicker to make a correct response and made more choices per session. They made fewer perseverative responses per loss, which could reflect an enhanced awareness of the increased likelihood of reward from a different choice. However, it could also indicate a greater aversion to loss/‘punishment’. Additionally, *Map2k7^+/−^* mice made more beam breaks per session than WT mice, suggesting that they were hyperactive throughout the task, a phenotype previously detected in these mice in the five-choice serial-reaction time task (5CSRTT) (Openshaw et al., 2017). We have also noted hyperactivity in this mutant strain in the open field arena (Openshaw et al., 2020), suggesting that *Map2k7^+/−^* mice are generally hyperactive, at least in a novel environment.

### Performance following Manipulation 1: switching reward/punishment contingencies of intermediate choices

Manipulation 1 consisted of switching the two more subtly different options in the task: Choice 1 with Choice 3. % Choice for 1 and 3 were reversed and mice re-acquired stable performance after just six sessions. Both *Map2k7^+/−^* and WT mice learned the switch to the same extent, with *Map2k7^+/−^* mice still performing slightly, but significantly, better once stable performance had been re-attained (less % Choice 4 and more % Choice 2). This ability of mice to notice a subtle change in reward–punishment contingencies is quite remarkable and informs us that C57Bl6 mice are clearly able to evaluate small differences in either the amount of reward they are receiving, the probability that they get rewarded/punished, the extent of the punishment when it occurs, or a combination of all three, and then alter behaviour to maintain optimal performance.

### Performance following Manipulation 2: switching reward/punishment contingencies of the most and least optimal choices

Manipulation 2 consisted of switching the location of Choices 2 and 4 (the most and least optimal options), as well as decreasing the punishment severity of Choices 3 and 4, so that punishment length increased linearly with reward amounts rather than exponentially. In the five sessions immediately following Manipulation 2, mice adjusted their % choices of the two that had been switched. All mice took longer to reach stable choice preference following this switch compared with Manipulation 1, and *Map2k7^+/−^* mice took more than twice as long as WTs to do this (*Map2k7^+/−^* mice, 30.25±4.17 sessions; WTs, 12.1±4.32 sessions). The overall increase in the number of sessions until re-achieving a stable choice pattern is likely to be due to the alteration of the punishment period, because it took the mice just six sessions following Manipulation 1, despite this switch being more subtly different, reward-value wise. By decreasing the punishment lengths for Choices 3 and 4, the difference between the most and least optimal options is much less, so that Choices 3 and 4 are less distinguishably ‘worse’ and therefore mice took longer to notice and alter their preference. If *Map2k7^+/−^* mice are especially sensitive to ‘punishment’, they may not have as much drive to avoid Choice 4 in its new location as they would if the punishment period had remained higher. Although WT mice took, on average, ∼5 extra days to learn Manipulation 2 compared to Manipulation 1, *Map2k7^+/−^* mice took ∼24 extra days. This suggests that punishment length, or possible reward achieved over time (which is directly affected by punishment length), has a large impact on their decision making, perhaps even more so than quantity or immediate probability of reward, and that this effect is stronger in *Map2k7^+/−^* than WT mice.

Interestingly, although mice were receiving negative feedback in the form of increased probability and length of punishment when selecting Choice 4, *Map2k7^+/−^* mice continued to persevere with selecting the now worst, previously best, option. This was very different from the behaviour observed following the less-extreme switching of contingencies (Manipulation 1). They showed over-reliance on the option that they had previously established to be most favourable and were more inflexible in their decision making than WT mice. In fact, *Map2k7^+/−^* mice behaved in a very similar way to that seen in patients with schizophrenia in the IGT study (Turnbull et al., 2006), in which task contingencies were also altered.

Evidence is accumulating that patients with schizophrenia are risk averse. In the balloon analogue risk task (BART), which is analogous in some respects to the IGT, but incorporates an overt risk element into every single ‘play’, patients with schizophrenia are abnormally wary of the potential for punishment (Reddy et al., 2014; Brown et al., 2015; Luk et al., 2019; Boka et al., 2020). This is also evident in IGT performance, where patients are especially sensitive to the frequency of punishment, rather than the magnitude. Patients robustly select the rare, large punishment, disadvantageous deck more frequently than controls, more than is the case with the frequent, small punishment, disadvantageous deck (Ritter et al., 2004; Shurman et al., 2005; Brown et al., 2015; Betz et al., 2019). Certainly, the *Map2k7^+/−^* mice were clearly different from WT mice in terms of their selection of the option with the most frequent punishment (Choice 4), even before the manipulations. The reasons for risk aversion in patients with schizophrenia are poorly understood. They may partially reflect worry and compromised rational thought, potentially linked to the experience of delusions (Freeman, 2016; So et al., 2020). Indeed, young people prone to delusions are more impaired than control subjects at the contingency-shifting stage of the modified IGT (Cella et al., 2009).

We do not interpret these data in terms of the *Map2k7^+/−^* mice being simply less cognitively flexible, and more perseverative, as they adapt just as well as WT mice to Manipulation 1. There seems to be a cognitive component probed by Manipulation 2 and not by Manipulation 1 that underlies their impaired performance. In a task interrogating ability to sustain attention, this strain shows similar accuracy to WT mice, but inattention, as evidenced by increased omission rates (Openshaw et al., 2016). However, we have not linked attentional impairment to the profound deficits reported here following Manipulation 2. The data presented in Table S1 show no evidence for increased omission rates in this gambling task (although once task acquisition is complete, omission rates are much lower than in the 5CSRTT, as expected, because the gambling task is not designed to probe attention – stimulus presentation time and allowed response time are much longer than in the 5CSRTT).

It is intriguing that mice showed dramatically increased reward collection latency after Manipulation 2, but not after Manipulation 1. The reasons for this are not clear. It may be that there is substantial salience attributed to these best and worst choices that leads to greater difficulty in decision making, and in carrying out the sequelae of making a response, once the outcome of selection of these choices becomes uncertain. The results on choice selection are very clearly different from prior predictions at this point, and it is possible that there is a heightened ambiguity about the expectation of reward, owing to the salience of the previously learned association. The increased latency effect is markedly exacerbated in *Map2k7^+/−^* mice. We speculate that there is a possibility that this reflects the association of sequence variations in the *MAP2K7* gene with risk for panic disorder as well as schizophrenia (Gregersen et al., 2016). Indeed, patients with panic disorder not only try to avoid high-risk options in the IGT (Giorgetta et al., 2012), but also take longer than controls to make their decisions (Beck et al., 1992; Lautenbacher et al., 2002; Kaplan et al., 2006) and experience reduced reward anticipation associated with their intolerance of uncertainty (Nelson et al., 2014).

### Manipulation 2 stable performance

Interestingly, when their performance was once again stable following Manipulation 2, the choice preference of the two groups of mice settled differently to the situation following Manipulation 1 (and at baseline): both groups of mice increased their preference for Choice 3 and Choice 4, WT mice decreased their preference for Choice 1, and *Map2k7^+/−^* mice instead decreased their preference for Choice 2. The increase in % Choices 3 and 4 is logical: they are no longer as disadvantageous as they were previously because of their decreased punishment length. The alteration in preference for Choices 1 and 2 by WT and *Map2k7^+/−^* mice is presumably in sacrifice for the increase in Choices 3 and 4; however, it is interesting that the groups picked different choices to sacrifice. *Map2k7^+/−^* mice perform worse as they have sacrificed a more optimal choice (Choice 3) in favour of the lesser optimal choice (Choice 4), which may reflect differences in reward valuation based on their previous knowledge of reward/punishment contingencies (Glimcher and Rustichini, 2004). It is worth noting that Choice 2 has longer punishment than Choice 1, so the reduced Choice 2 selection by *Map2k7^+/−^* mice may, again, reflect their preference to avoid longer punishment rather than taking into account reward quantity.

Here, we have demonstrated that mice hemizygous for genetic deletion of the JNK activator MKK7 show a highly specific performance deficit in a rodent touchscreen version of the human IGT. In particular *Map2k7*^+/−^ mice show an inability to adapt in a novel contingency-switching stage. These findings demonstrate a very precise role for MKK7–JNK signalling in flexibility of risk/reward assessment. The data imply that genetic variants affecting this molecular pathway may underlie impairment in this cognitive subprocess in schizophrenia and other neuropsychiatric disorders.

## MATERIALS AND METHODS

### Subjects

Mice heterozygous for *Map2k7* (*Map2k7^+/−^*) were produced as previously described (Sasaki et al., 2001) and backcrossed onto the C57Bl6/J strain. Twelve *Map2k7^+/−^* mice (six male, six female) and ten WT (five male, five female) littermates (from four different litters) were used. Mice were 15.2±0.04 weeks of age at the start of the study, with male mice weighing 31.1±0.49 g and female mice weighing 23.5±0.22 g, on average. All mice were pair housed in a temperature- and humidity-controlled room (21°C, 45-65% humidity) with a reversed 12-h light/dark cycle (lights off at 07:00). Mice were food restricted to 85-90% of their individual free-feeding weight and had *ad libitum* access to water throughout the experiment. Testing was carried out daily between 08:00 and 13:00, Monday to Friday and in accordance with the UK Home Office Animals (Scientific Procedures) Act 1986. Within genotype and sex restrictions, mice were randomly assigned to groups.

### rGT

The rGT was performed essentially as described previously (Thomson et al., 2021). In the rGT, mice are able to freely choose between four options that differ in magnitude and ratios of reward/punishment possibilities (Choices 1-4). Mice undergo up to 100 trials per daily, 30-min session. Overall, Choice 2 is the most advantageous option because it gives the highest amount of reward per unit time. Choice 1 is the next most advantageous option, followed by Choice 3, and Choice 4 is the least advantageous. Mice were trained on the rGT until they reached stable performance. Manipulation 1 then occurred (see below) until stable performance was re-attained, and then mice underwent Manipulation 2. A timeline of the rGT procedure is shown in Fig. 1A; the procedure and task contingencies largely followed those detailed by Zeeb et al. (2009) and the Touchscreen rGT User Manual (Campden Instruments), up to and including the Free Choice sessions.

Mice were tested in four identical touchscreen operant chambers within a sound- and light-attenuating box with a silent extractor fan (Campden Instruments). Mice were tested in the same operant box and were allocated a box randomly; however, no group (genotype or sex) exclusively carried out the rGT at the same time of day or in a particular operant box.

During initial box habituation/training, mice were introduced to the operant boxes and learnt to touch the screen to obtain reward. The stimulus (a white square) was displayed pseudorandomly in one of the four windows (grid positions 1, 2, 4 or 5) while the others remained blank. After 30 s, the stimulus was removed and 7 µl strawberry milkshake reward was delivered, with accompanied food dispenser light illumination.

The next sessions trained mice to touch the screen, but illumination of the main light for 5 s occurred if the mouse made an incorrect response (touching an unlit square) or missed the stimulus and did not respond (omission). As these sessions progressed, the length of time for which the stimulus is lit (stimulus duration) was decreased from 30 s to 10 s. Mice were moved on individually to the next stage when they achieved criteria of >80% accuracy and <20% omissions for two consecutive sessions. These stages of training took 49 sessions in total.

Next, mice were given four sessions to learn each of the reward/punishment contingencies. These sessions lasted for 30 min or 100 trials. The contingencies were counterbalanced across mice so that a quarter of mice were trained on one of four different combinations of locations of contingencies: A=Choice 1, 4, 2, 3; B=Choice 2, 1, 3, 4; C=Choice 3, 2, 4, 1; D=Choice 4, 3, 1, 2, in grid squares 1, 2, 4 and 5, respectively. The mice were forced to choose a particular square (i.e. only one square was lit at each trial) so that each mouse had equal prior exposure to each of the contingencies. A white square was presented in one of the four stimulus locations, and the limited hold (LH) and stimulus duration length was fixed at 10 s. Incorrect responses (not at the lit square) resulted in no action but were recorded. Failure to respond within the LH period was recorded as an omission, and then the stimulus was removed. A correct response at each square resulted in delivery of one to four amounts of 7 µl strawberry milkshake, or a ‘punishment’ timeout, in which the main light was turned on, stimuli were removed and the touched square flashed (0.2 s on and then 0.2 s off), according to the periods and frequencies shown in Fig. 1B.

Mice then moved onto the full task, where they had the option to pick any square at each trial. All other conditions remained similar to when they were forced to pick a choice, except all four of the stimulus squares appeared at each trial in grid positions 1, 2, 4 and 5. Mice were trained on this ‘free choice’ version until they reached stable performance, i.e. they were consistent in their choice pattern for 5 consecutive days, which took 13 sessions.

### Manipulation 1: switching locations of similar, intermediate return, reward/punishment contingencies

Choices 1 and 2 were the most optimal choices, and 3 and 4 were the least optimal, throughout training to stable performance. The position of the two most similarly optimal choices (1 and 3) were switched in Manipulation 1, making the contingency groups resemble the following: A=Choice 3, 4, 2, 1; B=Choice 2, 3, 1, 4; C=Choice 1, 2, 4, 3; D=Choice 4, 1, 3, 2, in grid squares 1, 2, 4 and 5, respectively (see Fig. 1B for an overview of each of the manipulations). Mice were tested on Manipulation 1 until they reached stable performance, which took six sessions.

### Manipulation 2: switching locations of most extreme reward/punishment contingencies

Mice were then subjected to a second manipulation, where Choices 2 and 4 (most versus least optimal) were switched. This was in addition to adjustment of the punishment period for Choices 3 and 4, which was decreased from 20 s and 40 s to 15 s and 20 s, respectively (Fig. 1B). The contingency groups for this manipulation resembled the following: A=Choice 3, 2, 4, 1; B=Choice 4, 3, 1, 2; C=Choice 1, 4, 2, 3; D=Choice 2, 1, 3, 4, in grid squares 1, 2, 4 and 5, respectively. By making the punishment less extreme for the disadvantageous options 3 and 4, it is possible to assess the sensitivity of mice to the punishment period length rather than sensitivity to reward amounts obtained per choice, or probability of reward/punishment. Choices 3 and 4 become less distinguishably ‘worse’, and therefore the amount of reward available to be achieved per unit time for the least optimal choices are now more similar to each other, making decisions more difficult. Mice were tested on Manipulation 2 until they reached stable performance, which took 56 sessions. The mice underwent one session per day for the first 28 sessions and then, to speed up learning as progress was so slow for the *Map2k7^+/−^* mice, two sessions per day for the second 28 days. Only the morning sessions were analysed when mice were run twice per day, as time of day may have an influence on task parameters. Hence 42 (morning) sessions were analysed, and the afternoon sessions were not analysed.

### Statistical analysis

Statistical analyses are described in each figure legend, or in the main text, and were carried out using Minitab^®^ 17 statistical software. Data are expressed either as mean±s.e.m., or as box (median and interquartile range) and whisker plots. Sample size was determined based on power analysis from our previous experience of effect size and variance with these tests, and was predicted to yield at least 80% power at *P*<0.05. All mice were included in all analyses, with the exception of two significantly atypical WT mice (who were removed from study at an early stage because of consistent abnormal repetitive behaviour – hyperactive rotational movements – that prevented them from completing the task).

## Supplementary Material

Supplementary information
