# Supplementary material for: The schizophrenia risk gene Map2k7 regulates responding in a novel contingency-shifting rodent touchscreen gambling task
Source: Dis Model Mech. 2022 Mar 11;15(3):dmm049310. doi: 10.1242/dmm.049310 (PMC8922023; doi:10.1242/dmm.049310)
Supplement: Supplementary information [file dmm-15-049310-s1.pdf]

Amphetamine administration

Choice Performance

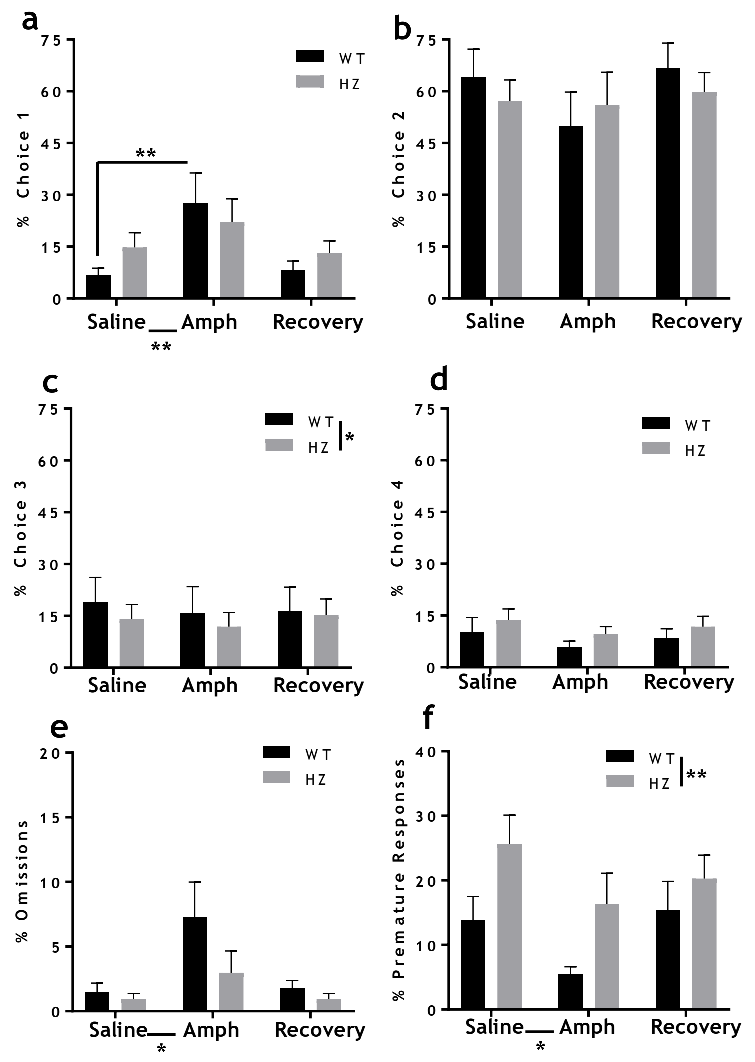

Fig. S1. An acute dose of amphetamine (1.5 mg/kg) slightly altered choice preference and other performance parameters in the rGT. Following amphetamine administration, preference for Choice 1 was increased (**a**), but Choice 2 (**b**), 3 (**c**), and 4 (**d**), were not significantly altered. % omissions were increased (**e**) and premature responses decreased (**f**) for all mice. Data represent the mean; error bars represent the SEM. Data was analysed by a three-way repeated measures ANOVA with treatment (saline or amphetamine) as a within subjects factor, genotype and gender as a between subjects factor. Tukey's post hoc analyses were carried out where appropriate: significant differences are indicated by *$p<0.05$, **$p<0.01$. Recovery is included on graph but not in analysis. Three-way ANOVA (with treatment (saline, amphetamine, recovery) as a within subjects factor, genotype and gender as a between subjects factor) were carried out on all the data before proceeding with comparison between saline and amphetamine in order to establish that proper recovery had occurred. According to Tukey's post-hoc analyses, there were no significant differences between saline treatment and recovery in any of the parameters. Therefore, the data from the saline group had no detectable carry-over effects from amphetamine on the second day of drug administration so saline was then only compared with amphetamine.

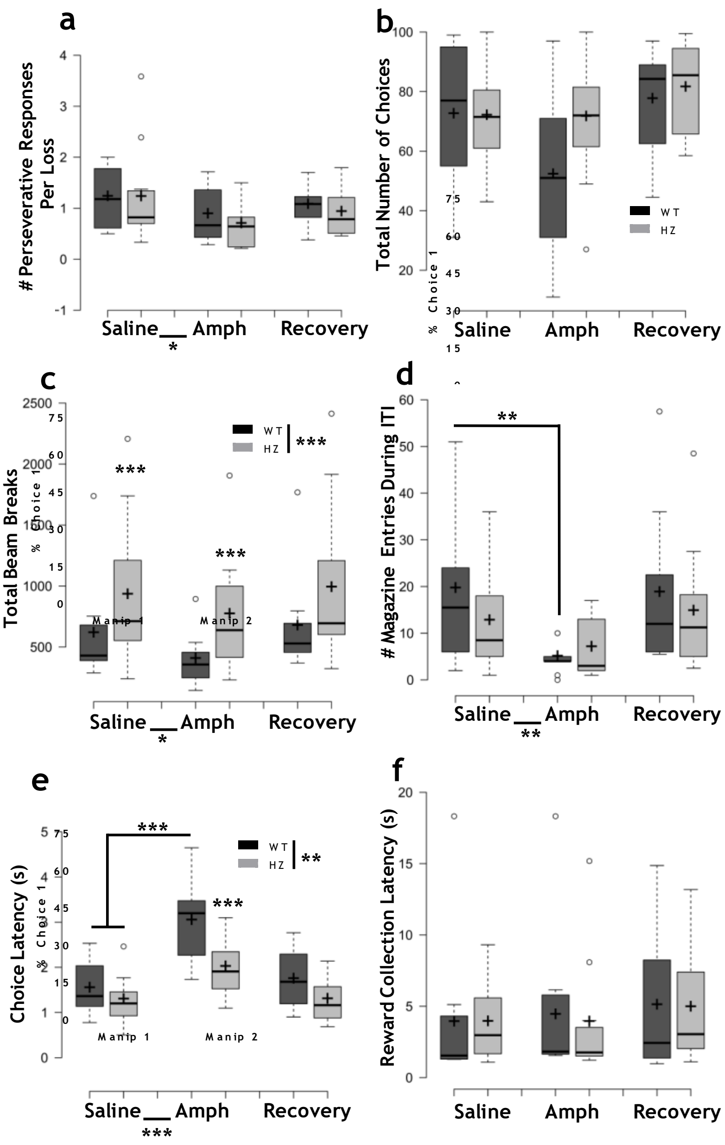

Fig. S2. An acute dose of amphetamine (1.5mg/kg) altered other performance / motivational parameters in the rGT. Following amphetamine administration, perseverative responses (**a**), total number of beam breaks (**c**) and magazine entries during the ITI (**d**) decreased, whereas choice latency (**e**) increased and total number of choices (**b**) and reward collection latency (**f**) remained the same. WT mice were particularly affected by amphetamine for choice latency (**e**) and magazine entries during the ITI (**f**). Data was analysed by a three-way repeated measures ANOVA with treatment (saline or amphetamine) as a within subjects factor, genotype and gender as a between subjects factor. Box plots: centre lines show the medians; box limits indicate the 25th and 75th percentiles as determined by R software; whiskers extend 1.5 times the interquartile range from the 25th and 75th percentiles, outliers are represented by dots; crosses represent sample means. Tukey's post hoc analyses were carried out throughout where appropriate: significant differences are indicated by **p<0.01, ***p<0.0001. Recovery is included on graph but not in analysis. Three-way ANOVA (with treatment (saline, amphetamine, recovery) as a within subjects factor, genotype and gender as a between subjects factor) were carried out on all the data before proceeding with comparison between saline and amphetamine in order to establish that proper recovery had occurred. According to Tukey's post-hoc analyses, there were no significant differences between saline treatment and recovery in any of the parameters. Therefore, the data from the saline group had no detectable carry-over effects from amphetamine on the second day of drug administration so saline was then only compared with amphetamine.

Table S1. Additional behavioural parameters at different stages of task performance

**Stage of task**	**WT**	***Map2k7* Hz**
Task acquisition (mid point)		
Correct choice latency	8.88 +/- 1.70	6.81 +/- 1.68
Reward collection latency	5.20 +/- 1.54	1.96 +/- 0.41 *
% omissions	13.6 +/- 4.8	11.2 +/- 5.3
No. perseverative responses	5.08 +/- 1.37	4.08 +/- 1.27
No. premature responses	8.50 +/- 2.45	8.67 +/- 2.36
No. of choices made	48.4 +/- 9.5	61.2 +/- 10.1
Magazine entries	16.2 +/- 2.7	23.3 +/- 6.0
Total beam breaks	506 +/- 33	660 +/- 69 *
Training stage on day	9.5 +/- 0.3	10.0 +/- 0.3
No.of mice at criteria 7d later #	3/11	5/11
Stable performance		
Correct choice latency	1.91 +/- 0.24	1.51 +/- 0.14 *
Reward collection latency	3.11 +/- 0.50	2.51 +/- 0.47
% omissions	3.55 +/- 1.79	2.48 +/- 0.97
No. of choices made	64.1 +/- 4.8	78.4 +/- 4.4 *
Magazine entries	17.2 +/- 3.4	14.7 +/- 3.4
Manipulation 1		
Correct choice latency	1.76 +/- 0.29	1.38 +/- 0.11 *
Reward collection latency	3.79 +/- 0.62	2.86 +/- 0.77
% omissions	3.05 +/- 1.83	1.70 +/- 0.61 *
No. of choices made	60.3 +/- 6.9	77.3 +/- 5.0 *
Magazine entries	11.6 +/- 2.2	11.2 +/- 1.4
Total beam breaks	675 +/- 79	892 +/- 105 *
Manipulation 2		
Correct choice latency	1.43 +/- 0.10	1.29 +/- 0.13
Reward collection latency	5.73 +/- 1.81	6.22 +/- 1.72
% omissions	1.23 +/- 0.41	1.41 +/- 0.90
No. of choices made	79.3 +/- 4.0	79.1 +/- 4.0
Magazine entries	19.7 +/- 4.9	12.1 +/- 1.9
Total beam breaks	748 +/- 99	973 +/- 134 *

- p<0.05 vs WT (Tukey). Data presented as mean+/- s.e.m., except for #

Table S2. Statistical analysis details for Figures

Figure	Paradigm	WT vs. Map2k7 Hz (Effect of Genotype)	Genotype x sex	Effect of sex	Notes
2	Choice 1	$F_{(1,80)}$= 0.07, p=0.795	$F_{(1,80)}$=0.18, p=0.677	$F_{(1,80)}$=10.82, p=0.001	F < M
2	Choice 2	$F_{(1,80)}$=6.73, p=0.011	$F_{(1,80)}$=0.34, p=0.564	$F_{(1,80)}$=0.00, p=0.970	
2	Choice 3	$F_{(1,80)}$= 1.33, p=0.237	$F_{(1,80)}$=1.51, p=0.222	$F_{(1,80)}$=36.91, p=0.000	M < F
2	Choice 4	$F_{(1,80)}$=15.85, p<0.0001	$F_{(1,80)}$=1.36, p=0.246	$F_{(1,80)}$=0.16, p=0.689	
2	Beam Breaks	$F_{(1,80)}$=461.80, p<0.0001	**$F_{(1,80)}$=50.77, p=0.000**	**$F_{(1,80)}$=17.05, p=0.000**	F > M, M WT < F Hz
3	Choice 1	$F_{(1,40)}$= 0.995, p=0.325	$F_{(1,40)}$=0.10, p=0.757	$F_{(1,40)}$=0.50, p=0.483	
3	Choice 2	$F_{(1,40)}$=12.47, p=0.001	$F_{(1,40)}$=8.55, p=0.006	$F_{(1,40)}$=0.08, p=0.783	M Hz < M WT
3	Choice 3	$F_{(1,40)}$= 0.53, p=0.478)	$F_{(1,40)}$=7.02, p=0.012	$F_{(1,40)}$=5.94, p=0.020	F > M F Hz > M Hz
3	Choice 4	$F_{(1,40)}$=56.28, p<0.001	$F_{(1,40)}$=6.48, p=0.015	$F_{(1,40)}$=0.90, p=0.348	M WT > M Hz
4	Choice 1	$F_{(1,80)}$ = 79.39, p<0.0001	$F_{(1,80)}$=0.06, p=0.811	**$F_{(1,80)}$=55.71, p=0.000**	M > F
4	Choice 2	$F_{(1,80)}$=61.05, p<0.0001	**$F_{(1,80)}$=12.27, p=0.001**	**$F_{(1,80)}$=209.58, p=0.000**	F > M F WT > M Hz
4	Choice 3	$F_{(1,80)}$=0.09, p=0.770	**$F_{(1,80)}$=18.53, p=0.000**	**$F_{(1,80)}$=31.35, p=0.000**	M > F M WT > F WT
4	Choice 4	$F_{(1,80)}$ = 4.38, p=0.039	$F_{(1,80)}$=0.40, p=0.529	**$F_{(1,80)}$=44.02, p=0.000**	M > F
5	Sessions to 70% optimal	F(1,20)=9.05, p<0.001	F(1,20)=0.68, p=0.419	F(1,20)=0.20, p=0.660	
5	Total choices BL	F(1,80)=19.16, p<0.001	F(1,80)=1.22, p=0.273	F(1,80)=0.30, p=0.584	
5	Total choices M1	F(1,65)=23.24, **p**<0.001	F(1,65)=**3.33, p**=0.073	F(1,65)=0.64, **p**=0.428	
5	Total choices M2	F(1,80)= 0.01, p=0.928	F(1,80)=3.25, p=0.075	**F(1,80)= 41.44, p=0.000**	F > M
5	Premature BL	F(1,80)=0.21, p=0.651	F(1,80)=15.08, p=0.000	**F(1,80)=12.89, p=0.001**	F > M F Hz > M Hz
5	Premature M1-1	F(1,65)=7.32, p=0.108	F(1,65)=0.86, p=0.359	F(1,65)=**32.26, p=0.001**	F > M
5	Premature M1-2	F(1,65)=2.77, p=0.105	F(1,65)=**10.7, p=0.002**	F(1,65)=**17.62, p=0.000**	F > M F WT > M WT
5	Premature M2-1	F(1,65)=1.51, p=0.226	F(1,65)=**15.3, p<0.001**	F(1,65)=**72.08 p<0.001**	F < M
5	Premature M2-2	F(1,80)=12.98, p=0.001	F(1,80)=2.04, p=0.158	F(1,80)=4.04, p=0.048	
5	Perseverative BL	F(1,80)=19.16, p<0.001	F(1,80)=1.07, p=0.304	F(1,80)=0.83, p=0.365	
5	Perseverative M1-1	F(1,65)=50.53, p<0.001	F(1,65)=**12.4, p=0.001**	F(1,65)=3.92 p=0.054	M WT<M Hz
5	Perseverative M1-2	F(1,65)=27.43, p<0.001	F(1,65)=4.23, p=0.047	F(1,65)=0.92 p=0.407	
5	Perseverative M2-1	F(1,65)=14.75, p<0.01	F(1,65)=0.00, p=0.960	F(1,65)=0.13 p=0.720	
5	Perseverative M2-2	F(1,80)=0.766 p=0.328	F(1,80)=0.440 p=0.528	**F(1,80)=13.50 p=0.000**	M > F
5	Choice Lat. BL	F(1,80)=34.96, p<0.001	F(1,80)=22.28, p<0.001	**F(1,80)=28.48, p<0.001**	M > F
5	Choice Lat. M1-1	F(1,65)=34.49, p<0.001	F(1,65)=**33.45,** p<0.001	**F(1,65)=65.45 p<0.001**	M > F
5	Choice Lat. M1-2	F(1,65)=24.66, p<0.001	F(1,65)=17.10, p<0.001	**F(1,65)=54.06 p<0.001**	M > F
5	Choice Lat. M2-1	F(1,65)=16.13, p<0.001	F(1,65)=1.33, p=0.256	**F(1,65)=123.2, p<0.001**	M > F
5	Choice Lat. M2-2	F(1,80)=7.42 p=0.009	F(1,80)=1.09 p=0.302	F(1,80)=6.92 p=0.011	
5	Reward Lat. BL	F(1,80)=2.26, p=0.137	F(1,80)=0.88, p=0.351	F(1,80)=0.04, p=0.842	
5	Reward Lat. M1-1	F(1,65)=8.11, p=0.007	F(1,65)=0.05, p=0.826	F(1,65)=**14.80, p=0.000**	F > M
5	Reward Lat. M1-2	F(1,65)=1.95, p=0.171	F(1,65)=1.09, p=0.303	F(1,65)=0.150 p=0.861	
5	Reward Lat. M2-1	F(1,65)=28.02, p<0.001	F(1,65)=0.65, p=0.424	F(1,65)=1.14 p=0.293	
5	Reward Lat. M2-2	F(1,80)=,0.360 p=0.548	F(1,80)=,0.370 p=0.547	**F(1,80)=,16.11, p=0.000**	M > F

BL = baseline

M1-1 = 1st 3 days after Manipulation 1

M1-2 = last 3 days after Manipulation 1

M2-1 = 1st 3 days after Manipulation 2

M2-2 = last 5 days after Manipulation 2

Disease Models & Mechanisms • Supplementary information
